# Procedural and one-year clinical outcomes of bioresorbable vascular scaffolds for the treatment of chronic total occlusions: a single-centre experience

**DOI:** 10.5830/CVJA-2016-033

**Published:** 2016

**Authors:** Erdem Özel, Baris Kilicaslan, Öner Özdogan, Ahmet Taştan, Ali Öztürk, Emin Evren Özcan

**Affiliations:** Cardiology Department, Tepecik Training and Research Hospital, İzmir, Turkey; Cardiology Department, Tepecik Training and Research Hospital, İzmir, Turkey; Cardiology Department, Tepecik Training and Research Hospital, İzmir, Turkey; Cardiology Department, Sifa University, İzmir, Turkey; Cardiology Department, Sifa University, İzmir, Turkey; Cardiology Department, Dokuz Eylul University, İzmir, Turkey

**Keywords:** bioresorbable vascular scaffold, chronic total occlusion, percutaneous coronary intervention

## Abstract

**Introduction:**

The bioresorbable vascular scaffold system (BVS) is the latest fully absorbable vascular therapy system that is used to treat coronary artery disease. The BVS has been used in different coronary lesion subsets, such as acute thrombotic lesions, bifurcation lesions, ostial lesions and lesions originating from bypass grafts. However, data about the use of BVS in chronic total occlusions (CTO) are limited. We report our BVS experience for the treatment of CTOs in terms of procedural features and one-year clinical follow-up results.

**Methods:**

An analysis was made of 41 consecutive patients with CTO lesions who were referred to our clinic between January 2013 and December 2014. A total of 52 BVS were implanted. An analysis was made of patient characteristics, procedural features [target vessel, BVS diameter, BVS length, post-dilatation rate, type of post-dilatation balloon, procedure time, fluoroscopy time, contrast volume, postprocedure reference vessel diameter (RVD), post-procedure minimal lesion diameter (MLD), type of CTO technique and rate of microcatheter use] and one-year clinical follow-up results [death, myocardial infarction, angina, coronary artery bypass graft (CABG), target-lesion revascularisation (TLR) and target-vessel revascularisation (TVR)]. Descriptive and frequency statistics were used for statistical analysis.

**Results:**

The mean age of the patient group was 61.9 ± 9.7 years, 85.4% were male, and 51.2 % had diabetes. Prior myocardial infarction incidence was 65.9%, 56.1% of the patients had percutaneous coronary intervention and 17.1% had a previous history of CABG. The procedure was performed via the radial route in 24.3% of the patients. The target vessel was the right coronary artery in 48.7% of the patients. Post-dilatation was performed on the implanted BVS in 97.5% of the patients, mainly by non-compliant balloon; 87.8% of the BVS were implanted by the antegrade CTO technique. Mean procedure time was 92 ± 35.6 minutes. Mean contrast volume was 146.6 ± 26.7 ml.

At one year, there were no deaths. One patient had lesionrelated myocardial infarction and needed revascularisation because of early cessation of dual anti-platelet therapy. Eleven patients had angina and five of them needed target-vessel revascularisation.

**Conclusions:**

BVS implantation appeared to be effective and safe in CTO lesions but randomised studies with a larger number of patients and with longer follow-up times are needed

## Introduction

Chronic total occlusion (CTO) is described as complete coronary vessel occlusion with a duration of three months or longer.^1^ Among patients diagnosed with coronary disease on angiography, the incidence of CTO lesions was between 20 and 30%.^2^ Successful CTO recanalisation provides better symptom control and survival outcome in the long term over failed revascularisation.^3^-^5^

According to recent guidelines, percutaneous recanalisation of CTOs should be considered in patients with expected ischaemia reduction in a corresponding myocardial territory and/or angina relief with a class 2a indication.^6^ Clinical outcomes of drugeluting stents (DES) are superior to bare-metal stents (BMS) in percutaneous revascularisation of CTOs.^7^,^8^

The bioresorbable vascular scaffold (BVS) (Absorb, Abbott Vascular, Santa Clara, CA, USA) is the latest fully absorbable vascular therapy system that is used to treat coronary artery disease. BVS has been tested in many randomised trials and provides some advantages over metallic stents because of its complete bioresorption process.^9^-^11^ BVS can facilitate the return of vessel vasomotor functions, reduce device thrombosis rates in the long term, make future surgical revascularisations more feasible and facilitate non-invasive imaging of the coronary arteries, since no metallic cage remains after two years.^12^,^13^

BVS has been used in different coronary lesion subsets, such as acute thrombotic lesions, bifurcation lesions, ostial lesions and lesions originating from bypass grafts. However, data on the use of BVS in chronic total occlusions (CTO) are limited. We report our BVS experience for the treatment of CTOs in terms of procedural features and one-year clinical follow-up results.

## Methods

Forty-one consecutive patients who underwent CTO revascularisation with one or two BVSs between January 2013 and December 2014 were analysed in the present study. A total of 52 BVSs were implanted. All patients were over 18 years old and had a diagnosis of stable angina pectoris. Lesions with a reference vessel diameter (RVD) of between 2.5 and 4 mm were included. Patients who had suffered from a myocardial infarction (MI) within one month of the procedure and patients who had left main coronary artery (LMCA) lesions or bifurcation lesions consisting of a side branch of over 2.5 mm were excluded. An informed consent for the procedure was obtained from each patient.

All CTO lesions were recanalised with dedicated CTO guide wires. After a mandatory pre-dilatation with an appropriate balloon, one or two BVS were implanted in the lesion. We did not use a strategy of hybrid stenting and no metallic stent was implanted in the lesions. Post-dilatation was performed with a compliant or non-compliant balloon after BVS implantation at the physician’s discretion if it was necessary.

Procedural features [target vessel, Japanese CTO score (J-CTO score), BVS diameter, BVS length, post-dilatation rate, type of post-dilatation balloon, procedure time, fluoroscopy time, contrast volume, post-procedure reference vessel diameter (RVD), post-procedure minimal lesion diameter (MLD), CTO technique and rate of microcatheter use] were analysed. Quantitative coronary angiography (QCA) measurements were used to assess RVD and MLD.

One-, three- and six-month, and one-year follow-up visits were made after each intervention. During routine visits, cardiovascular stress tests (treadmill exercise test or myocardial perfusion imaging test) were performed to diagnose the ischaemic situation associated with the intervention. Re-intervention and revascularisation were performed as needed. Rates of death, myocardial infarction (MI), angina, coronary artery bypass graft (CABG), target lesion revascularisation (TLR) and target-vessel revascularisation (TVR) were analysed.

## Statistical analysis

Measurement data were described as mean and standard deviation. Descriptive and frequency statistics were used for statistical analysis. The level of statistical significance accepted was 0.05. Data were analysed with the use of SPSS 17.0 software (SPSS, IBM, Chicago, USA).

## Results

Baseline patient characteristics and therapy at discharge are shown in Table 1. Thirty patients were treated by single BVS, and 11 patients were treated with two BVSs. Mean age was 61.9 ± 9.7 years, and 85.4% of the patients were male. Among our patient group, 51.2% had diabetes mellitus, 80.5% had hypertension and 46.3% had hyperlipidaemia. Renal function was within normal limits in all patients, 65.9% had prior MI, 56.1% had prior percutaneous coronary intervention (PCI) and 17.1% had prior CABG surgery; 24.3% of the procedures were performed by the radial route.

**Table 1:** Patient characteristics and therapy at discharge

*Patient characteristics*	*n = 41 patients (%)*
Age (years)	61.9 ± 9.7
Male gender	35 (85.4)
Diabetes	21 (51.2)
Hypertension	33 (80.5)
Hyperlipidaemia	19 (46.3)
Smoking	14 (34.1)
Chronic renal failure	-
Prior MI	27 (65.9)
Prior PCI	23 (56.1)
Prior CABG	7 (17.1)
Radial intervention	10 (24.3)
Lesion complexity (J-CTO score)	
Easy (J -CTO score of 0)	6 (14.6)
Intermediate (J-CTO score of 1)	23 (56)
Difficult ( J-CTO score of 2)	8 (19.5)
Very difficult (J-CTO score of ≥ 3)	4 (9.7)
Target vessel	
LAD	14 (34.1)
CX	7 (17)
RCA	20 (48.7)
Procedural success	41 (100)
Therapy at discharge	
ASA	41 (100)
Clopidogrel	35 (85.3)
Prasugrel	3 (7.3)
Ticagrelor	3 (7.3)
Statin	40 (97.5)
Beta-blocker	35 (85.3)

CABG: coronary artery bypass graft, CX: circumflex artery, J-CTO: Japanese CTO, LAD: left anterior descending artery, MI: myocardial infarction, PCI: percutaneous coronary intervention, RCA: right coronary artery.

**Fig. 1:**
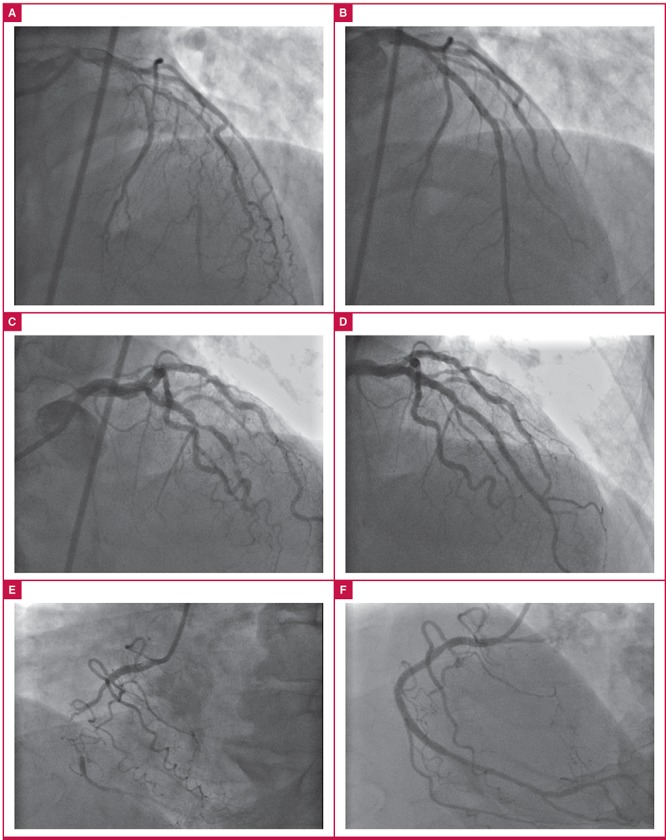
Coronary angiography showing recanalisation of chronic total occlusions. (A) Chronic total occlusion of the LAD from the proximal portion. (B) Successfull recanalisation of the LAD with two BVSs (3.5 × 28 mm and 3.0 × 28 mm) via antegrade approach. (C) Chronic total occlusion of the LAD from the mid portion. (D) Successfull recanalisation of LAD with two BVSs (3.5 × 18 mm and 3.0 × 28 mm ) via antegrade approach. (E) Chronic total occlusion of the RCA from the mid portion. (F) Successfull recanalisation of the RCA with one BVS (3.0 × 18 mm) via antegrade approach with the aid of a microcatheter.

Nearly half of the BVSs were implanted in the right coronary artery (RCA). Fourteen patients had lesions on the left anterior descending (LAD) artery and seven had lesions on the circumflex (CX) artery. Six patients had easy lesion complexity, 23 had intermediate complexity, eight had difficult, and four had very difficult complexity, according to the J-CTO score. Procedural success rate was 100%. Case examples are shown in Fig. 1.

All patients were treated with acetylsalicylic acid after the intervention. Additionally, 35 patients were treated with clopidogrel, three with ticagrelor and three with prasugrel. The rate of statin use was 97.5% among our patient group and betablocker use was 85.3%.

Mean BVS diameter and BVS length were 2.8 ± 0.29 and 25.6 ± 4.2 mm, respectively. Our post-dilatation rate was 97.5%, mainly by non-compliant balloon (NCB) (92.6%). Postprocedure RVD was 2.8 ± 0.25 mm and post-procedure MLD was 2.5 ± 0.25 mm. We performed CTO procedures mainly by the antegrade approach (87.8%). We used a microcatheter in 13 patients (31.7%).

Six patients had side branch occlusion and four had side branch narrowing. All of these patients were treated successfully by provisional stenting and final kissing balloon dilatation. Our procedure time was 92 ± 35.6 min, fluoro time was 20.2 ± 4.8 min and the mean value of contrast volume was 146.6 ± 26.7 ml (Table 2).

**Table 2:** Procedural characteristics

**	*n = 41 patients*
*Procedure*	*n = 52 BVS (%)*
BVS diameter, mm	2.8 ± 0.29
BVS length, mm	25.6 ± 4.2
Post-dilatation	40 (97.5)
Post-dilatation with NCB	38 (92.6)
RVD post-procedure, mm	2.8 ± 0.25
MLD post-procedure, mm	2.5 ± 0.25
Side-branch occlusion	6 (11.5)
Side-branch narrowing	4 (7.6)
CTO technique	
Antegrade	36 (87.8)
Retrograde	5 (12.1)
Microcathater use	13 (31.7)
Procedure time, min	92 ± 35.6
Fluoro time, min	20.2 ± 4.8
Contrast volume, ml	146.6 ± 26.7

BVS: bioresorbable vascular scaffold, CTO: chronic total occlusion, MLD: minimal lumen diameter, NCB: non-compliant balloon, RVD: reference vessel diameter.

At the end of one year, no death was observed. One patient had lesion-related MI and needed revascularisation because of early cessation of dual anti-platelet therapy. Eleven patients had angina and five of them needed TVR. Our TLR rate was 2.4% and TVR rate was 12.2% (Table 3).

**Table 3:** Clinical outcomes

*One-year outcome*	*n = 41 patients (%)*

Death	-
Myocardial infarction	1 (2.4)
Angina	11 (26.8)
Coronary artery bypass graft	-
Target-lesion revascularisation	1 (2.4)
Target-vessel revascularisation	5 (12.2)

## Discussion

Our study shows that BVS implantation in CTO lesions appeared to be effective and safe in terms of acute procedural and shortterm clinical follow-up results.

Theoretically, complete resorption of BVS, which is used for treating complex and calcific lesions, such as CTOs, seems to be advantageous. Lack of a metallic cage in these lesions can decrease the risk of restenosis, especially in the long term. Restoration of the vessel’s vasomotor functions may be easier with BVS implantation than with metallic stents.

Since patients with complex lesions such as CTOs have a greater risk for future CABG surgery, resorption of BVS in the treated segment may facilitate the performance of future graft anastomosis. However, in practice, the real effectiveness and safety of the use of BVS in CTO lesions are unclear and longterm clinical results are lacking. A few records and case reports have been published in the literature which analyse the role of BVS implantation in CTO lesions.^14^-^17^

The baseline characteristics of our patients were similar to those in previous BVS studies including patients with CTO lesions.^14^-^16^ In addition the cardiovascular risk profile of our patients was high and parallel with real-life records that investigate the effect of regular PCI procedures in CTO lesions.

Our mean BVS length was shorter than in previous studies.^14^,^15^ Most of our CTO lesions were within the treatable length of one BVS. Only 11 patients were treated with double BVS and no patients were treated with more than two BVSs. Although we had similar TLR rates and short-term clinical results with the BVS CTO studies, which included longer CTO lesions treated with a larger number of BVSs, studies with longer follow up beyond the resorption period of BVSs are needed in order to clearly determine the effect of scaffolds.

It is known that longer CTOs treated with a larger number of metallic stents have a greater risk of restenosis and worse clinical outcomes during follow up.^18^,^19^ Currently, we do not know whether complete resorption of the implanted scaffolds eliminates the risk of restenosis in long CTO segments in the long term.

Although BVS has thick struts (150 μm), a low crossing profile and less distensibility, our procedural success rate was 100% in our patient group. The main reasons for procedural success are effective dilatation after wiring the lesions, exact scaffold sizing with the aid of quantitative coronary angiography (QCA) measurements, and a high rate of post-dilatation. Lesion preparation before BVS implantation is a crucial factor, especially for CTO lesions.

Since a non-compliant balloon (NCB) reduces the procedure time compared with the compliant balloon,20 and is advised by many experienced centres,^21^,^22^ we preferred NCB for effective dilatation after pre-dilatation with lower-profile balloons. Also, a cutting balloon and rotablator can be used if needed. We performed post-dilatation in almost every lesion, mainly with a non-compliant balloon. We did not use balloons that had a size of more than 0.5 mm larger than the implanted BVS diameter. Post-dilatation with an inappropriate size of balloon can lead to fracture of the BVS in heavily calcific CTO lesions. We did not experience BVS fracture in our patient group.

Despite our high procedural success rate, implanting BVS in CTO lesions should be reserved for less complex CTO lesions, since experience is still limited. The J-CTO score, which characterises lesion complexity, could be a useful tool for decision making on indication. According to previous studies, CTO lesions that have a score of more than three (very difficult category) are associated with an unsuccessful procedure.^14^,^23^ The majority of our lesions were within the intermediate category according to the J-CTO score (56%). More complex lesions with a higher J-CTO score could affect procedural success and clinical outcomes.

During CTO procedures, jailing of the major side branches could be a problem, affecting the success of the procedure.^14^,^15^ Complete resorption of the BVS at the site of the bifurcation could lessen the effect of jailing and help return the side branch to normal vasomotor function. In our study, six side branch occlusions and four side branch narrowings were observed because of scaffold jailing. All of these lesions were treated with a provisional strategy with final kissing balloon dilatation.

Intravascular ultrasound (IVUS) and optical coherence tomography (OCT) are very valuable tools for evaluating the apposition of BVS during implantation.^12^ Not using IVUS or OCT is a limitation of our study but we had used QCA measurements for exact sizing of the BVS.

Since our study was non-randomised and lacked a control group, one should be cautious when interpreting the clinical data. A randomised study with a larger number of patients would be more valuable for evaluating the clinical outcomes. Our clinical follow-up results are too limited to evaluate the real clinical effectiveness of the use of BVS in CTO lesions. One year is a short follow-up period and cannot answer the question as to whether complete resorption of the BVS over two years will affect the clinical outcomes. However, short-term clinical results are promising.

## Conclusion

BVS implantation appears to be effective and safe in CTO lesions, according to our results, but randomised studies with a larger number of patients and a longer follow up are needed.

## References

[1] Di Mario C, Wener GS, Sianos G (2007). European perspective in the recanalisation of chronic total occlusions (CTO): consensus document from the EuroCTO Club.. EuroIntervention.

[2] Fefer P, Knudtson ML, Cheema AN (2012). The Canadian Multicenter Chronic Total Occlusions Registry. Current perspectives on coronary chronic total occlusions.. J Am Coll Cardiol Mar.

[3] Claessen BE, Dangas GD, Godino C (2011). Long-term clinical outcomes of percutaneous coronary intervention for chronic total occlusions in patients with vs. without diabetes mellitus.. Am J Cardiol.

[4] Joyal D, Afilalo J, Rinfret S (2010). Effectiveness of recanalization of chronic total occlusions: a systematic review and meta-analysis.. Am Heart J.

[5] Grantham JA, Jones PG, Cannon L, Spertus JA (2010). Quantifying the early health status benefits of successful chronic total occlusion recanalization: Results from the Flow-Cardia’s Approach to Chronic Total Occlusion Recanalization (FACTOR) Trial.. Circ Cardiovasc Qual Outcomes.

[6] Windecker S, Kolh P, Alfonso F (2014). 2014 ESC/EACTS Guidelines on myocardial revascularization: The Task Force on Myocardial Revascularization of the European Society of Cardiology (ESC) and the European Association for Cardio-Thoracic Surgery (EACTS). Developed with the special contribution of the European Association of Percutaneous Cardiovascular Interventions (EAPCI).. Eur Heart J.

[7] Colmenarez HJ, Escaned J, Fernandez C (2010). Efficacy and safety of drug-eluting stents in chronic total coronary occlusion recanalization: a systematic review and meta-analysis.. J Am Coll Cardiol.

[8] Patel MR, Marso SP, Dai D (2012). Comparative effectiveness of drugeluting vs. bare-metal stents in elderly patients undergoing revascularization of chronic total coronary occlusions: results from the National Cardiovascular Data Registry, 2005–2008.. J Am Coll Cardiol Cardiovasc Interv.

[9] Serruys PW, Chevalier B, Dudek D (2015). A bioresorbable everolimuseluting scaffold versus a metallic everolimus-eluting stent for ischaemic heart disease caused by de-novo native coronary artery lesions (ABSORB II): an interim 1-year analysis of clinical and procedural secondary outcomes from a randomised controlled trial.. Lancet.

[10] Ellis SG, Kereiakes DJ, Metzger DC (2015). Everolimus-eluting bioresorbable scaffolds for coronary artery disease.. N Engl J Med.

[11] Kimura T, Kozuma K, Tanabe K (2015). A randomized trial evaluating everolimus-eluting Absorb bioresorbable scaffolds vs. everolimus-eluting metallic stents in patients with coronary artery disease: ABSORB Japan.. Eur Heart J.

[12] Serruys PW, Ormiston JA, Onuma Y (2009). A bioabsorbable everolimuseluting coronary stent system (ABSORB): 2-year outcomes and results from multiple imaging methods.. Lancet.

[13] Dudek D, Onuma Y, Ormiston JA (2012). Four-year clinical follow-up of the ABSORB everolimus-eluting bioresorbable vascular scaffold in patients with de novo coronary artery disease: the ABSORB trial.. EuroIntervention.

[14] Wiebe J, Liebetrau C, Dörr O (2015). Feasibility of everolimus-eluting bioresorbable vascular scaffolds in patients with chronic total occlusion.. Int J Cardiol.

[15] Goktekin O, Yamac AH, Latib A (2015). Evaluation of the safety of everolimus-eluting bioresorbable vascular scaffold (BVS) implantation in patients with chronic total coronary occlusions: acute procedural and short-term clinical results.. J Invasive Cardiol.

[16] Ojeda A, Pan M, Romero M (2015). Outcomes and computed tomography scan follow-up of bioresorbable vascular scaffold for the percutaneous treatment of chronic total coronary artery occlusion.. Am J Cardiol.

[17] Rama-Merchan JC, Mattesini A, Dall'Ara G, Mario CD (2014). Chronic total occlusion successfully treated with a bioresorbable everolimus-eluting vascular scaffold.. Postepy Kardiol Interwencyjnej.

[18] Galassi AR, Brilakis ES, Boukhris M (2015). Appropriateness of percutaneous revascularization of coronary chronic total occlusions: an overview.. Eur Heart J.

[19] Ito T, Tsuchikane E, Nasu K (2015). Impact of lesion morphology on angiographic and clinical outcomes in patients with chronic total occlusion after recanalization with drug-eluting stents: a multislice computed tomography study.. Eur Radiol.

[20] Özel E, Taştan A, Öztürk A, Özcan EE, Uyar S, Şenarslan Ö (2015). What is better for predilatation in bioresorbable vascular scaffold implantation: a non-compliant or a compliant balloon?. Anatol J Cardiol.

[21] Caiazzo G, Kilic ID, Fabris E (2015). Absorb bioresorbable vascular scaffold: What have we learned after 5 years of clinical experience?. Int J Cardiol.

[22] Tamburino C, Latib A, Van Geuns RJ (2015). Contemporary practice and technical aspects in coronary intervention with bioresorbable scaffolds: a European perspective.. EuroIntervention.

[23] Morino Y, Abe M, Morimoto T (2011). Predicting successful guidewire crossing through chronic total occlusion of native coronary lesions within 30 minutes: the J-CTO (Multicenter CTO Registry in Japan) score as a difficulty grading and time assessment tool.. J Am Coll Cardiol Cardiovasc Interv.

